# IL-33-induced neuroimmune regulation in depression: A narrative review from molecular mechanisms to therapeutic potential

**DOI:** 10.1097/MD.0000000000047821

**Published:** 2026-02-28

**Authors:** Guiwei Wang, Xiaoming Zhang, Gang Duan, Shuping Yuan

**Affiliations:** a General Education College, Shandong Huayu University of Technology, Dezhou, China; b Adult Education Office, Dongying Vocational College, Dongying, China.

**Keywords:** depression, IL-33immune regulation, neural plasticity, neuroinflammation, therapeutic target

## Abstract

Depression is a common mental illness characterized by significant variability in treatment response and resistance to current antidepressant drugs and psychotherapy. Recent advances in neuroimmunology have highlighted the immune system’s crucial role in the pathogenesis of depression. This review explores the dual roles of interleukin-33 (IL-33) in neuroimmune regulation, neuroinflammation, and neuroplasticity, aiming to investigate its potential as a therapeutic target for depression. An integrative review of the literature was conducted, focusing on the molecular mechanisms of IL-33 in neuroinflammation and its impact on microglia, astrocytes, and the hypothalamic-pituitary-adrenal axis. Animal model studies and clinical evidence regarding IL-33 levels in depression were also analyzed. IL-33 exhibits both pro-inflammatory and anti-inflammatory functions and regulates key immune cells in the central nervous system, including microglia and astrocytes. It regulates neuroinflammation and improves neural plasticity, which is often impaired in depression. Clinical studies show decreased IL-33 levels in the blood and cerebrospinal fluid of depression patients, correlating with disease severity. IL-33 holds promise as a potential biomarker for depression and may serve as a therapeutic target. Recent therapeutic strategies targeting its receptor (suppression of tumorigenicity 2) and signaling pathways are under investigation, with early clinical trials focusing on anti-IL-33 receptor antibodies and signaling pathway inhibitors. However, challenges remain regarding immune-related side effects, and further clinical studies are needed to ensure the safety and efficacy of IL-33-targeted therapies.

## 1. Introduction

Depression, as one of the most prevalent mental disorders globally, can critically hinder individuals’ quality of life, and social acting.^[1]^ Although recent antidepressant medications, and psychotherapies exhibit efficacy in decreasing symptoms, specific variability in therapeutic responses, and the emergence of treatment resistance across various individuals remain prominent challenges. Evidence has determined that interindividual differences in antidepressant response are a primary factor causing inconsistent treatment outcomes.^[2]^ Besides, the efficacy of psychotherapeutic interventions varies critically among depression patients, especially with regard to treatment resistance, and long-term efficacy.^[2]^ Hence, the investigation of novel therapeutic targets, and mechanisms has been vital in recent depression article. Currently, developments in neuroimmunology have furnished new perspectives, implying the essential role of the immune system in the pathogenesis of depression.

Interleukin-33 (IL-33) is an emerging immune regulatory factor that has currently obtained increasing attention.^[3]^ Evidence suggests that the dual role of IL-33 in neuroimmune regulation, inflammatory response, and neuroplasticity positions it as a potential target in depression research.^[4]^ IL-33 plays a vital role in the immune system, and cna regulate the inflammatory response of the nervous system under the backdrop of suppressing tumor progression via its receptors (suppression of tumorigenicity 2 [ST2]).^[5]^ IL-33 is produced by astrocytes, and endothelial cells, while its receptor ST2 is markedly expressed on microglia, and astrocytes. The activation of these cells can cause the generation of pro-inflammatory cytokines, such as interleukin-1β (IL-1β), and tumor necrosis factor alpha (TNF-α), which increase neuroinflammation.^[4,6]^ Besides, IL-33 can be critical in boosting the expression of neurotrophic factors, and increasing neural plasticity, which may be vital in the onset, and progression of neurological, and psychiatric disorders, such as depression.^[6]^ In the context of regulating these pathways, it is assumed that IL-33 can powerfully improve depression triggered by neuroinflammation, especially in the depression subtype controlled by neuroinflammation. It is worth noting that although preclinical evidence has reported that IL-33 has integrative outlook, its employment in the treatment of depression is still in its infancy, and accessible research, and clinical evidence are restricted. Currently, there are still numerous uncertainties regarding the mechanism of action, enhancement of treatment strategies, and safety of IL-33 in depression. Thus, there is an urgent need to comprehensively explore the potential role of IL-33 in depression, and investigate its outlook, and challenges as a therapeutic target.

Given this context, we integrate recent literature from neuroimmunology, and psychiatry to explore the multifaceted role of IL-33 in depression. This includes its involvement in neuroimmune regulation, hypothalamic-pituitary-adrenal (HPA) axis regulation, and neuroplasticity, all of which support its potential as a therapeutic target for depression. By investigating the mechanisms through which IL-33 influences these pathways, we aim to provide a theoretical foundation for the development of targeted therapies. Additionally, we identify key challenges in translating IL-33-targeted therapies to clinical settings, and suggest directions for future research to address these challenges.

## 2. Materials and methods

### 2.1. Literature search

An extensive and systematic literature search was conducted to identify relevant studies discussing the role of IL-33 in depression. The search was performed using multiple databases, including PubMed, Scopus, and Web of Science, to collect peer-reviewed articles. The search was limited to articles published from January 1, 2000 to December 31, 2024. The keywords used in the search included “IL-33,” “depression,” “neuroinflammation,” “neuronal plasticity,” “HPA axis,” and “immune regulation.” Studies were selected based on their relevance to the topic, with a particular focus on the role of IL-33 in neuroinflammatory processes and depression, including animal models and human studies.

### 2.2. Inclusion, and exclusion criteria

The research in this review meets the following criteria: publication in peer-reviewed journals. Studied the role of IL-33 in neuroinflammation, depression, or related pathways such as HPA axis regulation, and neuroplasticity. Provide empirical data from preclinical (animal models) or clinical studies. The exclusion criteria are as follows: the article does not focus on IL-33 or its association with depression (or related psychiatric disorders). Literature published in non English languages. Opinion articles, comments, or editorials that do not present original research.

### 2.3. Data extraction

Data extraction is independently conducted by 2 reviewers (reviewer 1, and reviewer 2) to ensure accuracy, and decrease bias. The extracted data includes study design, sample size, population (preclinical or clinical), key findings, and conclusions related to the role of IL-33 in depression. Any disagreements between reviewers will be resolved through discussion. If consensus cannot be reached, the third reviewer (reviewer 3) will be consulted.

### 2.4. Quality assessment

Our aim is to integratively outline the role of IL-33 in depression by integrating existing preclinical, and clinical evidence. However, we acknowledge the inherent limitations of the included studies, such as variability in experimental design, sample size, and methodology. The results of this review should be explained in the context of these limitations, and further well-designed studies, especially human clinical trials, are needed to confirm the potential therapeutic significance of IL-33 in depression.

### 2.5. Scope of review

This review summarizes preclinical, and clinical studies on IL-33, and depression, providing a balanced discussion of molecular mechanisms, therapeutic potential, and translational correlations. Although most of the studies included in this review are preclinical studies, potential impacts on human health, and the treatment of depression were also discussed.

## 3. Findings

### 3.1. Biological features of IL-33

**Figure 1.**
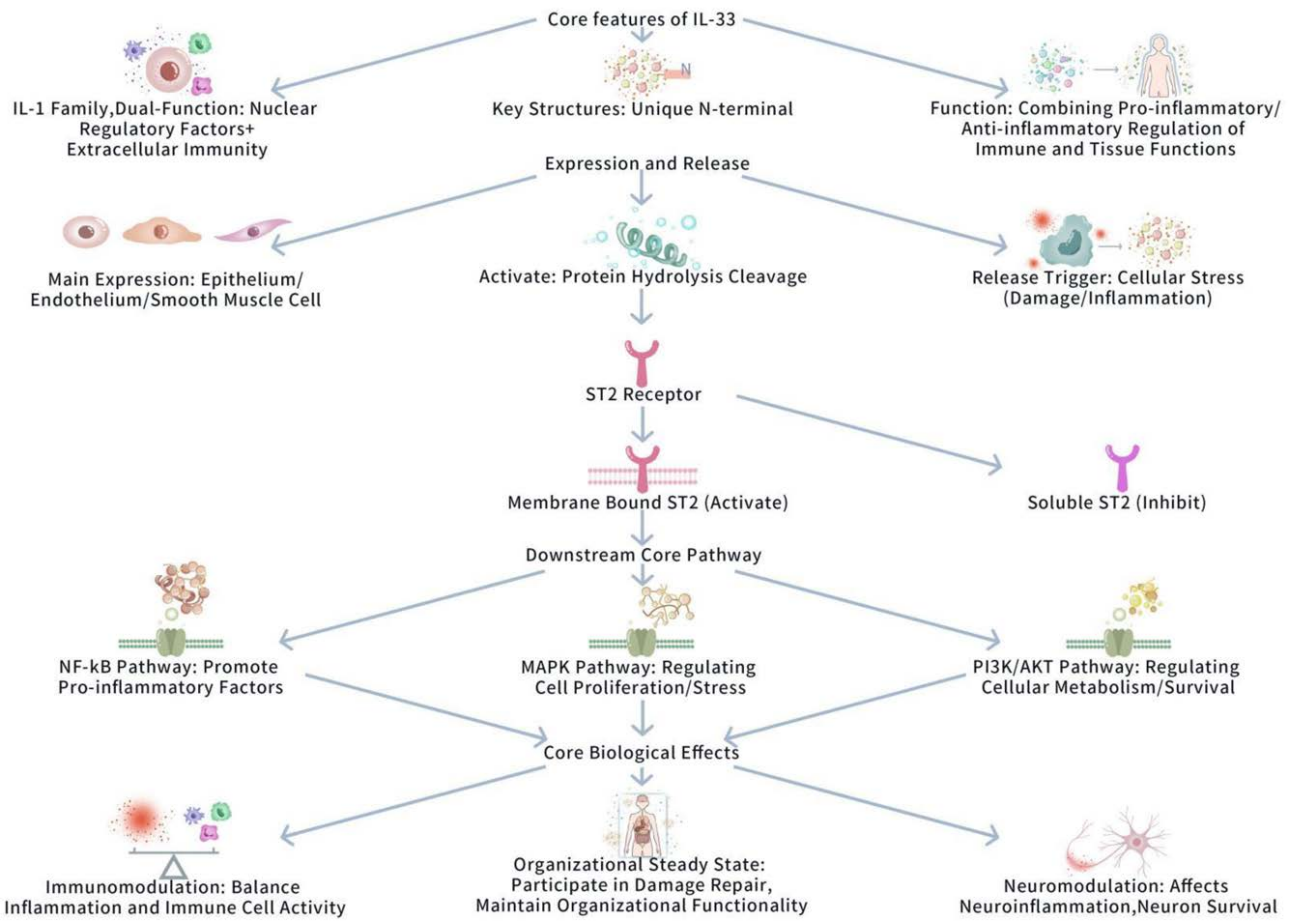
IL-33 mediated intracellular extracellular molecular cascade, and core regulatory mechanism. This figure illustrates the biological mechanism of action of interleukin-33 (IL-33), covering its core features (IL-1 family members, dual functions of nuclear regulation and extracellular immunity, unique N-terminal structure, dual immune and tissue function regulation effects of pro-inflammatory/anti-inflammatory), expression and release (mainly expressed in epithelial/endothelial/smooth muscle cells, cell stress triggered release, protein hydrolysis cleavage activation), receptor-mediated signal regulation (membrane-bound ST2 activation signal, soluble ST2 inhibition signal), as well as downstream Nuclear Factor - kappa B (pro-inflammatory cytokine production) The core pathways of mitogen-activated protein kinase (cell proliferation/stress regulation) and PI3K/Akt (cell metabolism/survival regulation) ultimately elucidate the core biological effects of IL-33 in immune regulation (balancing inflammation and immune cell activity), tissue homeostasis maintenance (participating in injury repair and ensuring tissue function), and neural regulation (affecting neuroinflammation and neuronal survival). IL-33 = interleukin-33, MAPK = mitogen-activated protein kinase, NF-κB = nuclear factor-kappa B, PI3K/Akt = phosphatidylinositol 3-kinase/protein kinase B, ST2 = suppression of tumorigenicity 2.

To systematically analyze the biological characteristics of IL-33, it is necessary to start from its molecular level basic properties – molecular structure determines functional characteristics, and family classification clarifies its positioning in the cytokine network. The 2 together constitute the core prerequisite for understanding the subsequent expression patterns, and signaling mechanisms of IL-33. Figure 1 displays IL-33 mediated intracellular extracellular molecular cascade, and core regulatory mechanism.

#### 3.1.1. Molecular structure, and classification

IL-33, a pivotal member of the IL-1 cytokine family, displays a obvious bifunctional nature, acting both as a nuclear regulator, and an extracellular cytokine inducing immune responses.^[7]^ Including roughly 270 amino acid residues, the molecular structure of IL-33 contains characteristic IL-1 family domains, in particular, demonstrated by 3 β-sheet folds, and an extended C-terminal tail.^[8,9]^ Similar to other IL-1 family members, IL-33 interacts with its receptor via its β-sheet structure, thus triggering following signaling pathways.^[9]^ However, IL-33 features a unique N-terminal region within its molecular structure, differentiating its functional regulation from IL-1β, and IL-18, and conferring it with obvious biological regulatory properties.^[7,8]^ Besides, IL-33 is grouped as a cytokine with pro-inflammatory, and anti-inflammatory functions in the IL-1 family. Of these, it can play a critical dual rolein managing immune responses, and staying tissue homeostasis.^[4,9]^ The IL-33 gene is found on human chromosome 9p24, and its expression is harshly regulated by various transcription factors. This precise regulatory mechanism determines that IL-33 performs its biological functions at the apt time, and place.^[4,9]^

#### 3.1.2. Expression, and production mechanisms

At the cellular expression level, IL-33 is primarily expressed by epithelial cells, endothelial cells, smooth muscle cells, and specific types of neurons. Notably, within the central nervous system (CNS), IL-33 expression is particularly abundant, especially localized in astrocytes, and certain neurons.^[10]^ This specific expression pattern implies a critical role for IL-33 in neuroimmune regulation.

In respect of production mechanisms, IL-33 displays unique features. It is worth noting that unlike other classical cytokines that count on the conventional protein production pathway, IL-33 can be secreted in response upon cell death or given that stress conditions.^[11,12]^ It is interesting that when cells suffer mechanical damage, oxidative stress, or inflammatory stimuli, IL-33 translocates from the nucleus to the cytoplasm, and is thereafter released into the extracellular environment.^[11,13]^ Besides, the activation mechanism of IL-33 is regulated by proteolytic cleavage, in which the cleaved IL-33 displays higher biological activity, enabling more positive interacting with its receptor, and activation of following signaling pathways.^[12]^ These findings not only confirm the critical role of IL-33 in cellular stress, damage, and inflammatory responses, but also highlight its unique production mechanisms.

#### 3.1.3. Receptors, and signaling pathways

The principal receptor for IL-33 is ST2, a critical member of the IL-1 receptor family.^[14]^ ST2 exists in 2 forms: the membrane-bound form ST2L, and the soluble form soluble suppression of tumorigenicity 2.^[15]^ Membrane – bound form ST2 (ST2L) binds IL-33 via its extracellular domain, thus triggering following signaling pathways^[16]^; oppositely, soluble suppression of tumorigenicity 2 binds IL-33, averting its interaction with ST2L, and thus regulating IL-33 activity.^[16]^

After interacting with ST2, IL-33 mainly triggers 2 central following signaling pathways: the nuclear factor-kappa B (NF-κB) pathway, and the mitogen-activated protein kinase (MAPK) pathway. Notably, IL-33 engages the NF-κB pathway via its receptor ST2, promoting the transcription of inflammatory genes, and promoting the expression of pro-inflammatory cytokines, such as TNF-α, and interleukin-6 (IL-6).^[15,17]^ This mechanism plays a vital role in the activation of immune cells, and inflammatory responses. For example, there is evidence reporting that the association with IL-33 to ST2 can activate the generation of pro-inflammatory cytokines via the NF-κB signaling pathway, which plays a vital role in immune, and inflammatory responses.^[15,17]^ Besides, under specific conditions, the nuclear localization domain of IL-33 can directly associate with NF-κB to hence regulate its activity, thus influencing the combined regulation of immune responses.^[15]^ Thus, the IL-33/ST2/NF-κB axis plays a critical role in immune, and inflammatory responses, especially in managing cytokine generation, and immune cell activation. Meanwhile, IL-33 can activate the MAPK signaling pathway via the ST2 receptor, including extracellular signal-regulated kinase, c-Jun N-terminal kinase, and p38 mitogen-activated protein kinase (p38 MAPK). These pathways are essential for managing cell proliferation, differentiation, and stress responses.^[18,19]^ In the nervous system, these MAPK signaling molecules are critical for managing neuronal survival, and function. For example, in the nervous system, the role of p38 MAPK has been widely assessed, reporting its involvement in neuroinflammation, neural injury, and repair processes.^[18,19]^

In addition, IL-33 can regulate the phosphatidylinositol 3-kinase (PI3K)/protein kinase B (Akt) signaling pathway, hence influencing cell metabolism, and survival ability. Of these, the PI3K/Akt pathway is essential for cell survival, proliferation, and metabolic regulation, especially in neurons, as it improves metabolic stability by managing glycolysis, cell growth, and survival.^[20,21]^ It is interesting that the activation of these signaling pathways not only displays impacts in immune cells, but also has a critical impact on neuroinflammation, and neuroplasticity within neurons.^[22]^ In particular, the regulation of the PI3K/Akt pathway can regulate the physiological functions of neurons by influencing neuroinflammatory responses, neuroprotection, and neuroplasticity.^[21]^

Overall, IL-33 triggers NF-κB, MAPK, and PI3K/Akt pathways via its receptor ST2, thus managing various biological mechanisms in immune, and neural cells. In the biological regulatory network, the complicated interactions of these signaling pathways play a core role in inflammatory responses, and critically impact the function, and pathological state of the nervous system, implying the critical role of IL-33.

### 3.2. Neuroimmune regulation, and depression

#### 3.2.1. Overview of the neuroimmune system

Recent studies have gradually demonstrated that the CNS is not a conventional immune privileged site, but rather has complicated neuroimmune regulatory mechanisms.^[23,24]^ In this complicated network, microglia, and astrocytes hold the position of the main immune cells of the CNS, being vital in observing neural homeostasis, removing neuronal debris, and regulating inflammatory responses.^[23]^ As intrinsic immune cells of the CNS, microglia can react to various pathological stimuli via morphological, and functional changes, thus being vital in neuroinflammation, and neurodegenerative diseases.^[24]^ Conversely, astrocytes singly take part in neuroimmune regulation by supporting neuronal function, managing neurotransmitter concentrations, and staying the integrity of the blood–brain barrier.^[23,24]^ It is worth noting that cytokines, chemokines, and cell adhesion molecules can be central to the regulatory processes of neuroimmune interactions, especially in the outbreak, and development of neuroinflammation. For example, pro-inflammatory cytokines, such as IL-1β, and TNF-α play a core role in regulating inflammatory responses, with IL-1β being particularly significant in causing ongoing endothelial cell adhesion responses.^[25]^ Besides, the balance of anti-inflammatory cytokines, such as interleukin-10 (IL-10), and transforming growth factor-beta (TGF-β) is comparably essential, as they play a decisive role in suppressing neuroinflammation, and boosting neural tissue repair.^[25]^

#### 3.2.2. The role of neuroinflammation in depression

An increasing number of studies have demonstrated that neuroinflammation plays a critical role in the pathogenesis of depression.^[26,27]^ Particularly, inflammatory markers, such as C-reactive protein, IL-6, and TNF-α in the peripheral blood, and brain of depression patients are critically increased.^[26,27]^ These inflammatory cytokines impact neuronal function, and plasticity via numerous mechanisms, causing emotional disorders, cognitive impairments, and behavioral changes.^[26,27]^

The influence of neuroinflammation on neuronal function is mainly revealed in several perspectives. One of them is the imbalance of neurotransmitter metabolism, where inflammatory cytokines can suppress serotonin synthesis, and metabolism via various pathways, thus influencing emotional regulation. In particular, inflammatory response triggers indoleamine 2,3-dioxygenase, which turns tryptophan to kynurenine instead of being applied for serotonin synthesis, causing serotonin depletion.^[28,29]^ Besides, cytokines, such as IL-6, and TNF-α can also impact other neurotransmitters by modifying the transport, and discharge mechanisms of dopamine, and norepinephrine, thus inducing depressive symptoms.^[28,29]^ These findings highlight that inflammatory cytokines suppress serotonin synthesis, and increase depressive symptoms by impairing the function of other neurotransmitters. Thus, managing inflammatory response may become a new method for treating depression. For decreased neural plasticity, inflammation suppresses the expression of neurotrophic factors, such as BDNF (BDNF), thus influencing neuronal survival, and synaptic plasticity. BDNF plays a vital role in the nervous system by interacting with TrkB receptors, triggering numerous signaling pathways, boosting neuronal growth, survival, and synapse formation.^[30,31]^ There is evidence to suggest that BDNF is essential for the formation, and maintenance of synapses, and decreased BDNF expression is tightly linked to cognitive, and emotional disorders.^[30]^ For example, immune responses in inflammation-related diseases can cause a decrease in BDNF levels, which have a limited impact on neuronal survival, and synaptic plasticity, thus increasing the risk of emotional disorders, such as depression.^[31,32]^ These findings highlight the significance of BDNF in neural progression, synaptic plasticity, and emotion regulation, and determine that the inhibition of its function by inflammatory responses may play an essential role in the pathogenesis of various neurological, and psychiatric disorders.

In addition, the dysfunction of the HPA axis is a critical factor in the pathophysiology of depression. The study highlights that overabundant activation of the HPA axis can cause an increase in cortisol production, which may be triggered by a decrease in core corticosteroid receptor function, thus impairing the limited response loop on the HPA axis.^[32]^ Besides, inflammatory cytokines, especially IL-1β, are believed to disrupt HPA axis function, causing abnormal stress responses, and hence aggravation of depressive symptoms. In particular, the dawn cortisol response in depression patients is negatively linked to the expression of IL-1β, implying a potential interaction between the immune system, and HPA axis dysfunction.^[33]^ Chronic overactivation of the HPA axis can impact the immune system, and may also damage brain structures linked to emotion regulation, and memory, such as the hippocampus, hence inducing depressive symptoms.^[33]^ There is evidence to suggest that inflammatory cytokines impact the stability, and function of neural networks via various mechanisms regarding the influence of neuroinflammation on cognitive function, and behavioral changes. For example, cytokines, such as IL-6, and TNF-α can alter neuronal function or drive neuronal apoptosis, causing synaptic loss, and following neural network transforming, and functional interruption.^[34,35]^ This type of neuronal apoptosis is tightly linked to synaptic reduction, which enhances depressive symptoms, and may impact treatment outcomes, and prognosis, especially when applying anti-inflammatory drugs or undergoing nerve regeneration interventions.^[35]^

**Figure 2.**
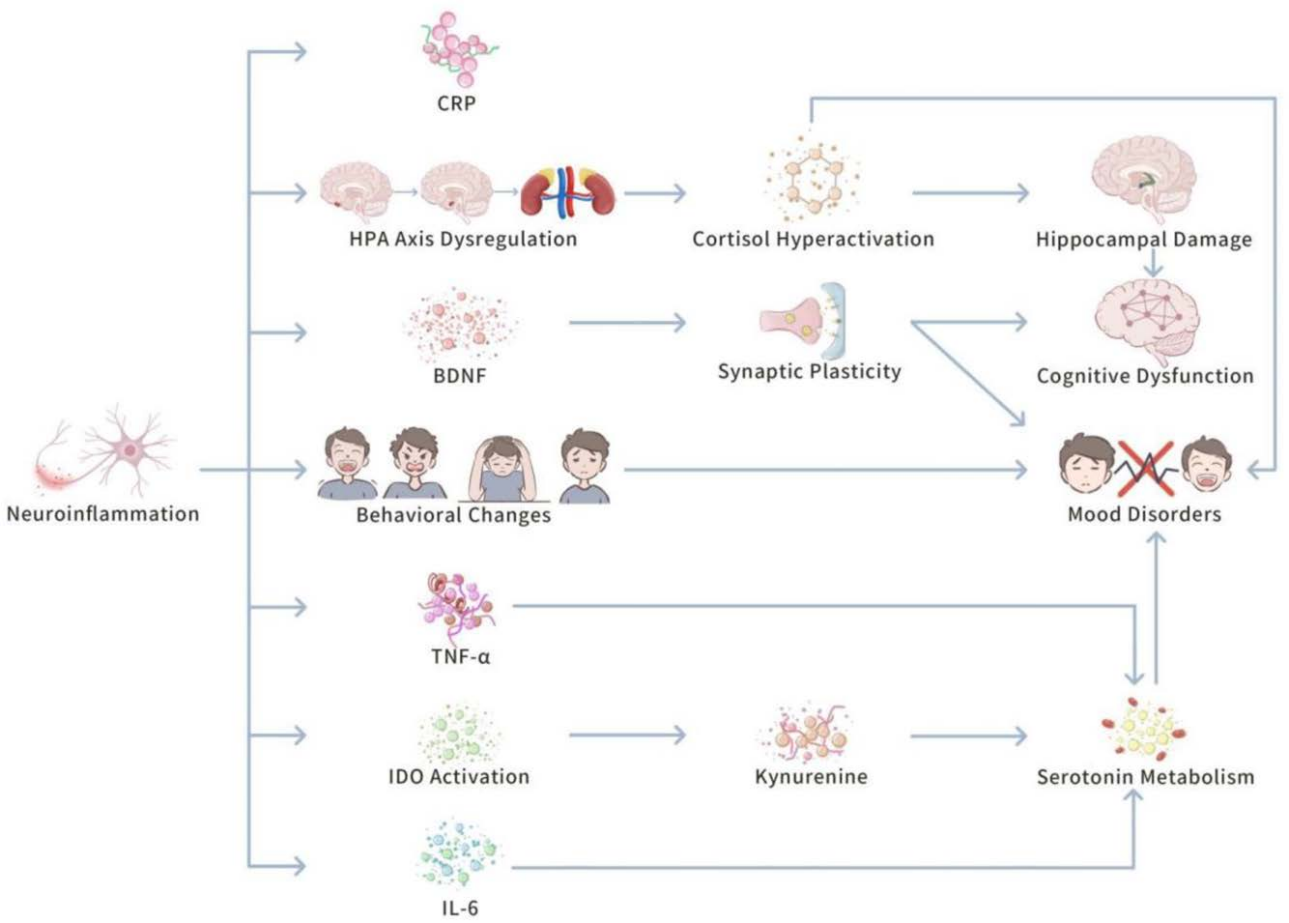
The role of neuroinflammation in the pathogenesis of depression. This figure displays how neuroinflammation inducing the pathogenesis of depression via numerous pathways. First, neuroinflammation triggers increased levels of inflammatory markers (e.g., CRP, IL-6, and TNF-α). These inflammatory factors, via activation of IDO, cause abnormal tryptophan metabolism, and generation of KYN, which in turn suppresses serotonin synthesis, disturbing mood regulation, and worsening depressive symptoms. At the same time, neuroinflammation also suppresses the expression of BDNF (BDNF), causing hindered synaptic plasticity, which in turn influences neuronal survival, and function, ultimately causing cognitive dysfunction, and mood disorders. Besides, by impacting the HPA axis, neuroinflammation causes cortisol overgeneration, causing hippocampal damage, which in turn enhances cognitive impairment, and depressive symptoms. BDNF = brain-derived neurotrophic factor, CRP = C-reactive protein, HPA axis = hypothalamic-pituitary-adrenal axis, IDO = indoleamine 2,3-dioxygenase, IL-6 = interleukin-6, KYN = kynurenine, TNF-α= tumor necrosis factor-α.

Overall, the neuroimmune system can critically influence the pathogenesis of depression via complicated cytokine networks, and signaling pathways. From the regulation of neurotransmitter metabolism to the maintenance of neural plasticity, and the modulation of the HPA axis, neuroinflammation inducing depressive symptoms, and pathological processes at numerous levels. Thus, therapeutic strategies targeting neuroinflammation hold promise for supplying new perspectives, and methods for clinical interventions in depression. Figure 2 displays the role of neuroinflammation in the pathogenesis of depression.

### 3.3. Molecular mechanisms of IL-33 in beuroinflammation

**Figure 3.**
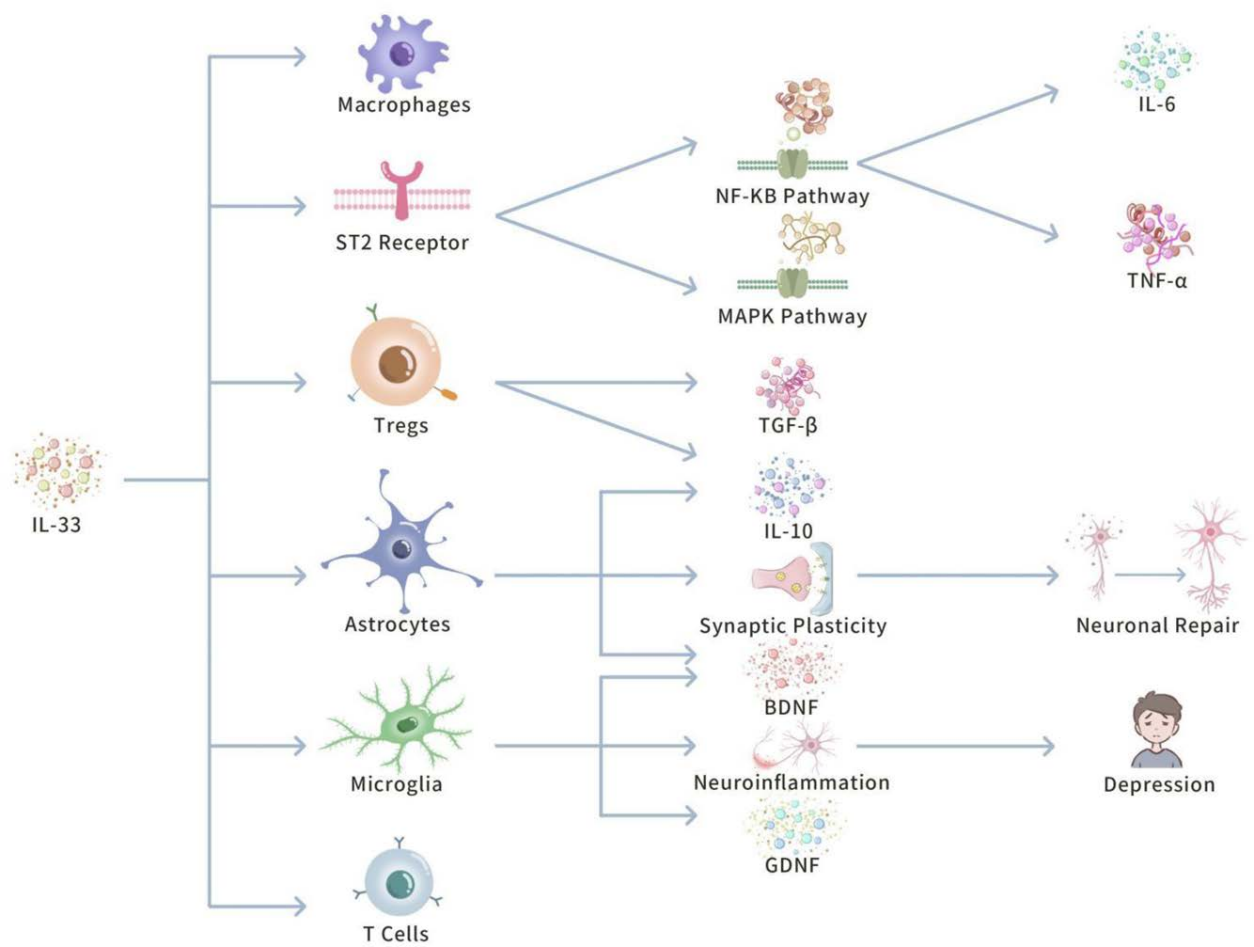
Molecular mechanism of IL-33 in neuroinflammation, and its double role. This figure illustrates the complicated mechanism by which IL-33 regulates immune cell, and neuronal cell functions in neuroinflammation via ST2 receptors. IL-33 performs a pro-inflammatory role in acute inflammation by interacting with ST2 receptors to activate the NF-κB, and MAPK signaling pathways, promoting pro-inflammatory cytokines, such as IL-6, and TNF-α, and boosting the activation of immune cells, such as Tregs, and macrophages. Role. Besides, IL-33 facilitates the generation of anti-inflammatory factors, such as IL-10, and TGF-β in regulatory Tregs, suppresses immune hyperactivation, and displays anti-inflammatory impacts. Meanwhile, IL-33 also regulates microglia, and astrocytes in the CNS, boosting their shift to an anti-inflammatory phenotype, and production of neurotrophic factors, such as BDNF, and GDNF, reducing neuroinflammation, protecting neurons, and boosting synaptic plasticity, and nerve repair. These mechanisms of action are important for neuroinflammation-related diseases, such as depression, and other neurodegenerative disorders, in terms of protection, and repair. BDNF = brain-derived neurotrophic factor, CNS = central nervous system, GDNF = glial cell line-derived neurotrophic factor, IL-10 = interleukin-10, IL-33 = interleukin-33, MAPK = mitogen-activated protein kinase, NF-κB = nuclear factor-kappa B, ST2 = suppression of tumorigenicity 2, TGF-β = transforming growth factor-beta, Tregs = regulatory T cells.

The unique double function of IL-33 enables it to play complicated, and multifunctional roles in the immune response of the nervous system, both by increasing inflammatory responses to react to acute pathological stimuli, and under the backdrop of suppressing overabundant immune activation to maintain tissue homeostasis, and balance. Figure 3 illustrates this double effect.

#### 3.3.1. Double functions of IL-33: pro-inflammatory, and anti-inflammatory

The dual role of IL-33 in neuroinflammation has determined as a central focus of research, implying its intricate regulatory functions in both pro-inflammatory, and anti-inflammatory processes. Evidence has implied that IL-33 binds to the ST2 receptor, thus triggering the NF-κB, and MAPK signaling pathways, which boost the amplification of the inflammatory response. This mechanism entails the induction of pro-inflammatory cytokines, such as IL-6, and TNF-α.^[4]^ Besides, the expression levels of IL-33 are linked to the activation of immune cells, especially being vital in the regulation of Tregs, and macrophages.^[4]^

Conurrently, the anti-inflammatory functions of IL-33 in immune regulation have obtained critical attention. Particularly, IL-33 improves the differentiation, and function of regulatory T cells (Tregs), and drives the generation of anti-inflammatory cytokines, such as IL-10, thus suppressing the expression of pro-inflammatory cytokines, and positively curbing the persistence, and expansion of the inflammatory response. For example, the expression of the IL-33 receptor ST2 is critically upregulated in Treg cells, which can improve the accumulation of Treg cells in inflammatory environments, and enhances their immunosuppressive functions.^[4,36]^ Hence, research highlights that IL-33, via its receptor ST2, not only inducing signal transduction in regulatory Tregs, but also critically increases their suppressive functions, especially within the intestinal, and other nonlymphoid tissues. ST2-expressing Treg cells secrete IL-10, and TGF-β, positively suppressing the hyperactivation of other immune cells, preserving immune tolerance, and averting overabundant inflammatory responses.^[36]^

This double functionality enables IL-33 to flexibly regulate inflammatory responses given that various pathological conditions. For example, during the acute phase of inflammation, the pro-inflammatory actions of IL-33 aid in the rapid response to pathogen infections, and tissue damage, thus producing a protective effect.^[7]^ Conversely, in the context of chronic inflammation or autoimmune diseases, the anti-inflammatory functions of IL-33 become particularly important, contributing to prevent the overabundant expansion of the inflammatory response, and preserve tissue homeostasis.^[4]^ This functional versatility renders IL-33 a pivotal factor in managing neuroinflammatory responses, with its role in psychiatric disorders, such as depression undergoing considerable attention.^[4,6]^

#### 3.3.2. Interaction between IL-33, and microglia

Microglia, as the main immune cells within the CNS, play an essential role in observing the neural environment, and responding to inflammatory signals. IL-33 regulates the activation state, and functionality of microglia by interacting with the ST2 receptor on their surface, thus producing a critical impact in neuroinflammation.^[37,38]^ Particularly, IL-33 facilitates the polarization of microglia towards an anti-inflammatory phenotype, increases the generation of anti-inflammatory cytokines, and lowers the expression of pro-inflammatory cytokines.^[37]^ This mechanism not only relieves neuroinflammation, but also positively protects neurons from inflammatory damage.^[38]^

In the pathophysiology of depression, the overabundant activation, and continuous pro-inflammatory phenotype of microglia increase neuroinflammatory responses, causing neuronal dysfunction, and diminished synaptic plasticity.^[39]^ However, IL-33 displays potential anti-inflammatory, and neuroprotective impacts by managing the activation state of microglia, and suppressing their pro-inflammatory functions.^[6,40]^ For example, animal model evidences have confirmed that overexpression of IL-33 critically lowers the pro-inflammatory response of microglia, decreases the levels of inflammatory markers, and ameliorates depressive-like behaviors.^[39,40]^

In addition, the interaction between IL-33, and microglia performs a critical role in the support, and repair functions of neurons. Literature highlights that IL-33 triggers microglia to secrete neurotrophic factors, such as brain-derived neurotrophic factor (BDNF), and glial cell line-derived neurotrophic factor, which are essential for neuronal survival, functional recovery, and synaptic plasticity.^[40]^ Particularly, BDNF performs an essential role in boosting neuronal growth, and the repair of neural circuits, which has been confirmed to critically increase synaptic plasticity, and cognitive functions in depression patients.^[41]^ Conurrently, by regulating the function of microglia, IL-33 relieves neuroinflammation, and supports neuronal repair, thus producing a positive impact on the emotional, and cognitive recovery of depression patients.^[6,40]^

#### 3.3.3. Interaction between IL-33, and astrocytes

Astrocytes perform a multitude of supportive, and regulatory functions within the CNS, including the regulation of neurotransmitter concentrations, maintenance of blood–brain barrier integrity, and support of neuronal survival, and synaptic plasticity.^[42]^ IL-33 regulates the functionality, and activity of astrocytes by interacting with the ST2 receptor on their surface, thus influencing neuroinflammation, and synaptic plasticity.^[43]^

Researches have confirmed that IL-33 improves the production of anti-inflammatory cytokines, such as IL-10, by astrocytes while suppressing the expression of pro-inflammatory cytokines.^[4,18]^ Besides, IL-33 increases the supportive functions of astrocytes towards neurons, boosting the generation of neurotrophic factors, and synaptic repair.^[9,18]^ For example, A Focapplied Systematic Review by Zharichenko and Njoku determined that supplementation with IL-33 critically enhanced the anti-inflammatory phenotype of astrocytes, lowered the release of inflammatory cytokines, and facilitated the survival of neurons, and the restoration of synaptic plasticity.^[4]^ Astrocytes plays a vital role in synaptic plasticity. IL-33, by regulating the functions of astrocytes, facilitates the formation, and pruning of synapses, thus preserving the stability, and plasticity of neural networks.^[44,45]^ This mechanism has critical implications for the cognitive functions, and emotional regulation in depression patients. Particularly, IL-33-induced production of neurotrophic factors, such as BDNF by astrocytes increases synaptic plasticity in neurons, facilitates the formation of new synapses, and repairs recent synapses, thus critically increasing the cognitive functions, and emotional states of depression patients.^[44,45]^

Overall, IL-33 can display its key role in neuroinflammation by regulating the functions of microglia, and astrocytes. This tiered regulatory mechanism can helps slow neuroinflammatory responses, and can also facilitate the restoration of synaptic plasticity, thereby providing a novel theoretical foundation, and underlying targets for the treatment of neuropsychiatric disorders, especially depression.

### 3.4. Regulation of the HPA Axis by IL-33

**Figure 4.**
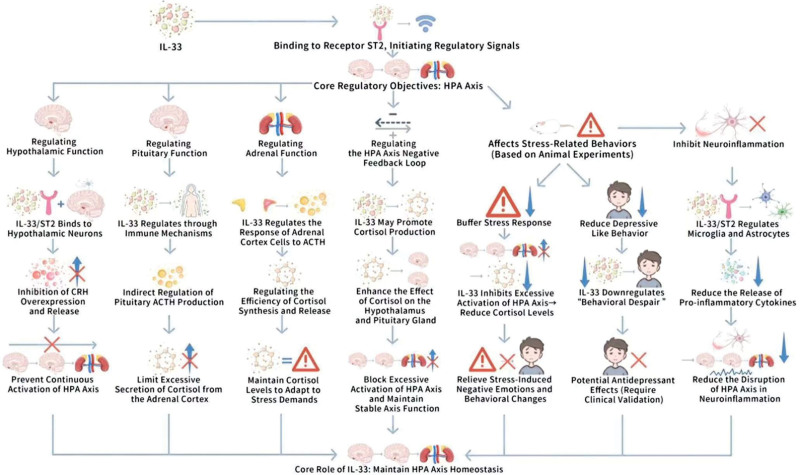
Mechanisms of IL-33 regulation of the HPA axis. This figure systematically illustrates the core mechanism by which IL-33 regulates HPA axis function through its receptor ST2, and its associated roles in stress response, depression related pathology, and neuroinflammation. The basic function of the HPA axis is first clarified in the figure: under normal conditions, the HPA axis mediates the body’s stress response through a cascade reaction of “hypothalamic secretion of CRH → pituitary secretion of ACTH → adrenal cortex secretion of cortisol”; Under pathological conditions (such as depression related), excessive activation of the HPA axis leads to sustained elevation of cortisol, which in turn triggers neurotoxicity (such as hippocampal neuron damage), immune suppression, and persistent inflammation, exacerbating depressive symptoms (emotional abnormalities, cognitive impairment, etc). IL-33/ST2 inhibits the excessive release of hypothalamic CRH, indirectly regulates pituitary ACTH production, and regulates the response of adrenal cortex cells to ACTH, thereby avoiding excessive activation of the HPA axis, and excessive secretion of cortisol at the level of the “hypothalamus pituitary adrenal” tertiary structure; IL-33 can increase its negative feedback effect on the hypothalamus (inhibiting CRH), and pituitary (inhibiting ACTH) by promoting cortisol production, further maintaining HPA axis homeostasis; Based on animal experimental evidence, IL-33 exerts antidepressant like effects by inhibiting HPA axis overactivation, and buffering stress-induced negative emotions, alleviating “behavioral despair” in forced swimming test (FST), and tail suspension test (TST); IL-33/ST2 regulates the activation status of microglia, and astrocytes, decreases the release of pro-inflammatory factors such as IL-1β, and TNF-α, and alleviates the disruption of HPA axis in neuroinflammation. ACTH = adrenocorticotropic hormone, CRH = corticotropin-releasing hormone, FST = forced swimming test, HPA axis = hypothalamic-pituitary-adrenal axis, IL-33 = interleukin-33, ST2 = suppression of tumorigenicity 2, TST = tail suspension test.

As a critical immunomodulatory factor, IL-33 not only performs a central role in the immune system, but also impacts the function of the CNS via various pathways. IL-33 is tightly linked to the regulation of the HPA axis, and performs a critical role in the pathogenesis of stress, and mood disorders. Figure 4 depicts mechanisms of IL-33 regulation of the HPA axis.

#### 3.4.1. The role of the HPA axis in stress response, and depression

The HPA axis forms an essential endocrine regulatory system by which the organism responds to external stressors. The activation of this axis commences with the hypothalamus secreting corticotropin-releasing hormone (CRH), which triggers the anterior pituitary to release adrenocorticotropic hormone (ACTH), thereafter inducing the adrenal cortex to secrete cortisol.^[46]^ As the principal hormone of the stress response, cortisol regulates the organism’s response to stress via various pathways, including metabolic regulation, immunoinhibition, and neural function modulation.^[47,48]^ Furthremore, a study has revealed the neural circuitry of the HPA axis, stressing its pivotal role in the stress response.^[47]^ Another publication outlines the regulatory mechanisms of corticotropin-releasing factor, and urocortin (Ucn) in stress, and adaptation, hence implying the significance of the HPA axis in stress adaptation.^[48]^

In the pathological mechanisms of depression, the HPA axis is regularly distinguished by hyperactivation, causing constantly increased cortisol levels.^[32]^ Dysregulation of the HPA axis is tightly linked to various symptoms of depression, including mood disturbances, cognitive dysfunction, and behavioral alterations.^[49]^ Chronic increased cortisol displays neurotoxic impacts on neurons, especially in the hippocampus, where such toxicity may induce neuronal apoptosis, and a decrease in neuroplasticity, thus worsening the pathological progression of depression.^[32]^

Notably, dysregulation of the HPA axis can not only hinder the acting of the immune system, but can also improve the persistence of inflammatory responses, thus worsening neuroinflammation, and neural dysfunction.^[32]^ Thus, preserving normal HPA axis function is essential for emotional stability, and proper cognitive function, while its dysregulation forms a core component in the pathogenesis of depression. Evidence has determined that chronic stress interacts with HPA axis dysregulation, and immune system activation to facilitate inflammatory responses, and that HPA axis dysregulation is tightly linked to the onset of depression.^[50]^ Chronic stress is tightly linked to hyperactivity of the HPA axis, which not only facilitates the outbreak of neuroinflammation but may also cause cognitive dysfunction, thus turning into one of the critical mechanisms given thatlying depression.^[32]^

In addition, overabundant activation of the immune system, especially the release of pro-inflammatory cytokines, such as IL-1β, and TNF-α, is tightly linked to HPA axis dysfunction, and the progression of depression.^[50]^ The release of these cytokines not only enhances neuroinflammation but may also hence disturb HPA axis regulation, creating a vicious cycle that intensifies depressive symptoms.

#### 3.4.2. Molecular mechanisms by which IL-33 regulates the HPA axis

IL-33 displays critical regulatory impacts on the HPA axis via its receptor ST2. The association with IL-33 to the ST2 receptor triggers following signaling pathways, thus influencing the function of the HPA axis, and the stress response.

##### 3.4.2.1. Regulation of hypothalamic function by IL-33

As a member of the IL-1 family, IL-33 is extensively expressed in the CNS, especially in essential regions, such as the hypothalamus. via its interaction with the ST2 receptor, IL-33 regulates neuroimmune responses, and performs a pivotal role in various neurodegenerative diseases, and immune regulation.^[10,43]^ Literature highlights that the IL-33/ST2 axis not only serves as an alarm in immune responses, but also influences the function of the HPA axis by managing neuronal activity.^[10]^ Particularly, IL-33 interacts with hypothalamic neurons via the ST2 receptor to regulate the expression, and production of CRH, thus suppressing overabundant CRH release, and averting continuous activation of the HPA axis.^[51]^ This mechanism highlights the potential role of IL-33 in managing stress responses, and related disorders.

##### 3.4.2.2. Regulation of pituitary function by IL-33

IL-33 performs a critical role in immune responses via its receptor ST2, and has been determined to regulate various metabolic disorders.^[52]^ regarding the regulation of ACTH production, evidences have confirmed that ACTH in the anterior pituitary is chiefly regulated by CRH, and limited feedback from cortisol.^[53]^ The role of IL-33 may indirectly impact ACTH production via immune regulatory mechanisms, thus impacting cortisol production levels from the adrenal cortex.^[52]^ This mechanism supports maintain normal ACTH production, and prevent overabundant elevation of cortisol levels, thus fulfilling an essential role in immune responses, and metabolic homeostasis.

##### 3.4.2.3. Regulation of adrenal function by IL-33

IL-33 regulates adrenal function by regulating the response of adrenal cortical cells to ACTH, thus influencing the synthesis, and release of cortisol, and playing an critical role in preserving HPA axis homeostasis. Literature has determined that ACTH in the adrenal cortex responds to stress by boosting the synthesis of glucocorticoids, with cortisol functioning as a central product of the HPA axis that is essential for managing metabolism, immunity, and behavior.^[54]^ Besides, IL-33 may impact cortisol release by regulating the action of ACTH, thus boosting apt cortisol production to signify normal HPA axis function, and stress adaptation.^[55]^ Thus, IL-33 potentially performs a critical immunoregulatory role in regulating the stress response of the HPA axis, and preserving endocrine balance.

##### 3.4.2.4. Regulation of response loops

It is noteworthy that IL-33 has been determined performs a critical role in the limited response loops of the HPA axis, possibly helping in the maintenance of HPA axis homeostasis by managing cortisol production, and action. Cortisol displays its impacts on the hypothalamus, and pituitary gland via numerous limited feedback loops, suppressing the production of CRH, and ACTH, thus regulating the stress response.^[56,57]^ In addition, cortisol has immunomodulatory impacts, capable of suppressing the production of pro-inflammatory cytokines, such as IL-1, IL-6, and TNF-α, thus decreasing inflammatory responses. As a critical immunomodulatory factor, IL-33 may increase cortisol production, thus reinforcing this limited feedback loop, and averting overabundant activation of the HPA axis.^[58]^ Overall, IL-33 causes preserving the balance of the HPA axis in immune regulation, and stress responses, averting its dysfunction.

#### 3.4.3. The relationship between IL-33, and stress-related behaviors

##### 3.4.3.1. Regulation of stress responses

IL-33 plays a crucial role within the immune system, and literature has confirmed that IL-33 can regulate stress responses in the context of managing metabolic, and signaling pathways across various immune cells. In animal models of stress, interestingly, the expression of IL-33 is critically upregulated, reflecting that it may play a role in buffering the limited emotions, and behavioral changes induced by stress.^[11]^ Literature has revealed that IL-33 buffers stress-driven limited emotions, and behavioral alterations under the backdrop of suppressing overabundant activation of the HPA axis, thus uncoupling the continuous production of cortisol. Aberrant activation of the HPA axis is regularly tightly linked to mood disorders, such as depression, and the upregulation of IL-33 may supply a protective effect by managing cortisol production.^[32]^ The regulatory impacts of IL-33 extend beyond immune responses, as it also performs a critical role in the regulation of emotions, and behaviors. Evidence has uncovered that by regulating immune, and endocrine responses, IL-33 can exert positive impacts on stress-induced emotional, and behavioral changes.^[11]^ However, it is important to note that while these findings are promising, the majority of the cited evidence comes from preclinical animal studies, and there is currently no direct clinical evidence from human studies to support these findings.

##### 3.4.3.2. Promotion of depressive-like behaviors

Evidence has determined that overexpression or exogenous supplementation of IL-33 can critically increase depressive-like behaviors in stress-induced animal models. Particularly, in forced swim tests, and tail suspension tests (TST), animals exhibit critically enhanced activity levels, signifying that IL-33 has potential therapeutic impacts in alleviating depressive symptoms.^[59]^ Besides, related research has found that IL-33 lowers “behavioral despair” in mice during forced swim test, and tail suspension test, likely being vital in antidepressant therapy.^[6]^ These findings support the potential of IL-33 as a novel therapeutic agent for antidepressant treatments. However, it is crucial to highlight that these results are based on animal models, and there is no direct clinical evidence available from human studies to corroborate these effects in humans.

##### 3.4.3.3. Inhibition of neuroinflammation

IL-33, via its receptor ST2, displays important immunomodulatory impacts within the nervous system, especially being vital in the activation states of microglia, and astrocytes. IL-33 can regulate the functions of these glial cells, thus suppressing neuroinflammatory responses. For example, evidence has determined that the IL-33/ST2 signaling pathway performs a critical role in the activation of microglia, hence influencing the progression of neuroinflammation.^[60]^ Besides, 1 publication outlines how IL-33 mitigates neuroinflammatory responses by managing the activation of astrocytes, and microglia.^[5]^ Additionally, research by Cao et al highlights that IL-33/ST2 signaling functions not only in endothelial cells, but also plays a crucial role in the activation of microglia, and regulation of neuroinflammation.^[60]^ While these findings are supported by preclinical studies, it is important to note that direct evidence from clinical human studies is lacking. Further research in human populations is needed to investigate whether the mechanisms observed in animal models are applicable to human conditions, especially in neuroinflammatory diseases such as depression.

### 3.5. IL*-33, and neuroplasticity*

**Figure 5.**
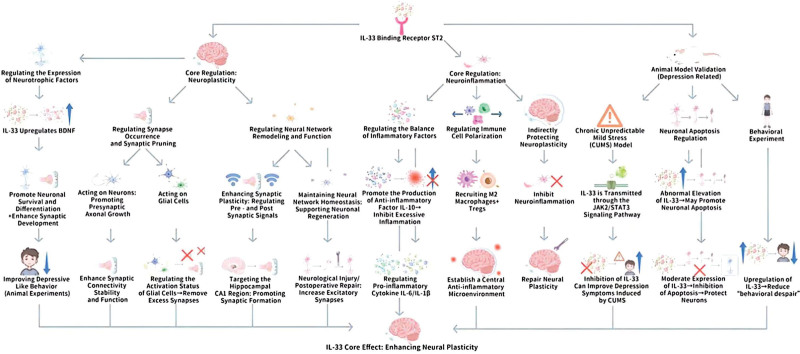
Mechanisms of IL-33 in the regulation of neuroplasticity, and neuroinflammation. This figure displays Mechanisms of IL-33 in the regulation of neuroplasticity, and neuroinflammation. IL-33 activates multidimensional regulatory mechanisms by binding to receptor ST2, being vital in balancing neuroplasticity, and neuroinflammation. On the one hand, it upregulates the expression of BDNF (BDNF), promoting neuronal survival, synapsis, and LTP in the hippocampal CA1 region. At the same time, it regulates the phagocytosis of ECM by microglia, and the activation of astrocytes, achieving synaptic pruning, and neural network optimization, enhancing synaptic plasticity, and improving cognitive memory in an experience dependent manner, and alleviating age-related synaptic dysfunction; On the other hand, it improves the production of anti-inflammatory factor IL-10 through the MyD88 dependent pathway, and JAK2/STAT3 signaling, recruits M2 macrophages, and regulatory Tregs to construct an anti-inflammatory microenvironment, inhibits the release of pro-inflammatory factors such as IL-6, and IL-1β, and affects depressive like behavior by regulating neuroinflammation in chronic stress models. However, its effect is bidirectional-moderate expression can protect neurons, while abnormal elevation may improve neuronal apoptosis, and exacerbate pathological processes. BDNF = brain-derived neurotrophic factor, CUMS = chronic unpredictable mild stress, ECM = extracellular matrix, IL-10 = interleukin-10, IL-6 = interleukin-6, IL-1β = interleukin-1β, JAK2/STAT3 = Janus kinase 2/signal transducer and activator of transcription 3, LTP = long-term potentiation, M2 = M2 macrophages, Tregs = regulatory T cells, ST2 = suppression of tumorigenicity 2.

As a central inflammatory mediator, IL-33 has been observed to regulate neuronal, and glial cell function, and take part in the stabilization, and transforming of neural networks. Thus, determining the mechanism of IL-33’s role in neuroplasticity is essential for unraveling adaptive changes in the nervous system, and the pathogenesis of related diseases. Figure 5 depicts the mechanism of IL-33 in the regulation of neuroplasticity, and neuroinflammation.

#### 3.5.1. Fundamental concepts of neuroplasticity

Neuroplasticity refers to the CNS’s ability to adapt structurally, and functionally, including neuronal survival, synaptogenesis, and synaptic pruning, coupled with the transforming of neural networks. This mechanism performs an essential role in learning, memory, and adaptation to environmental changes.^[61]^ Neuroplasticity involves not only alterations in the strength of connections between pre-, and postsynaptic neurons, but also the generation of new synapses, and the elimination of superfluous ones, thus implying the enhancement, and efficient acting of neural networks.^[62,63]^

In the study of depression, a decrease in neuroplasticity is deemed a critical component of its pathophysiological mechanisms. Literature has stressed that depression patients regularly exhibit decreased neuronal numbers, decreased synaptic density, and lowered expression levels of neurotrophic factors (e.g., BDNF) in brain regions, such as the hippocampus, and prefrontal cortex. These alterations cause dysfunctional neural networks, and mood disturbances, but impaired cognitive functions, and abnormal behaviors.^[41,64]^ It is interesting that increasing neuroplasticity has been considered as a central objective in antidepressant trestments for depression. Both pharmacological agents, and environmental factors can improve neuroplasticity, thus repairing damaged neural circuits, and helping in the restoration of brain function.^[41,64]^

#### 3.5.2. Regulation of neuroplasticity by IL-33

IL-33 performs multifaceted roles in managing neuroplasticity, chiefly by influencing the expression of neurotrophic factors (such as BDNF), and participating in the mechanisms of synaptogenesis, and synaptic pruning.

##### 3.5.2.1. IL-33’s influence on the expression of neurotrophic factors

BDNF is an essential regulator of neuroplasticity, involved in neuronal survival, differentiation, and the modulation of synaptic plasticity. Evidences have determined that IL-33 can upregulate BDNF expression levels, thus boosting neuronal survival, and synaptogenesis.^[65,66]^ In animal models, overexpression of IL-33 critically enhanced BDNF levels, ameliorated depressive-like behaviors, and increased synaptic density in neurons.^[67]^ This mechanism highlights that IL-33 indirectly improves the restoration, and enhancement of neuroplasticity by managing the expression of neurotrophic factors.

##### 3.5.2.2. Mechanisms of IL-33 in synaptogenesis, and synaptic pruning

Synaptogenesis, and synaptic pruning are core processes of neuroplasticity that identify the fine-tuning, and enhancement of neural networks. IL-33 interacts with neurons, and glial cells via its receptor ST2 to regulate the structural, and functional perspectives of both presynaptic, and postsynaptic sites. Particularly, IL-33 facilitates axonal growth in presynaptic neurons, and dendritic spine formation in postsynaptic neurons, thus increasing the stability, and functionality of synaptic connections.^[65]^ Besides, IL-33 is involved in the synaptic pruning mechanism by regulating the activation states of microglia, and astrocytes, thus boosting the clearance of unnecessary synapses, and strengthening the structure of neural networks.^[65]^ This regulatory mechanism not only causes preserving the health of neural networks, but also performs a critical role in adapting to environmental changes, and learning new information.

#### 3.5.3. IL-33, and neural network transforming

##### 3.5.3.1. Role of IL-33 in neural network stability, and transforming

IL-33 facilitates the stability, and adaptive transforming of neural networks by managing the functions of neurons, and glial cells. Promotion of neurotrophic factor production: IL-33 triggers neurons, and glial cells to secrete BDNF, and other neurotrophic factors, supporting neuronal survival, and synaptogenesis, thus increasing the connectivity strength, and functionality of neural networks.^[68]^

Enhancement of synaptic plasticity: IL-33 enhances synaptic plasticity by managing presynaptic, and postsynaptic signaling, enabling neural networks to more positively adapt to new information, and environmental changes. Literature has stressed that IL-33, produced by astrocytes in the hippocampal circuitry, regulates synaptic plasticity, and maintains synaptic transmission between neurons.^[65]^ Particularly, IL-33 regulates synaptic functions in the hippocampal CA1 region, promoting synapse formation, and functional recovery, thus increasing cognitive functions, and spatial memory.^[69]^ In addition, IL-33 relieves postoperative cognitive impairment in aged mice by increasing the number of excitatory synapses, and increasing cognitive functions linked to synaptic plasticity.^[70]^ These findings stress the central role of IL-33 in synaptic plasticity, especially in the adaptive modulation of neural networks in response to environmental changes.

Notably, given that conditions of neural injury or inflammation, IL-33 aids in restoring the functional integrity of neural networks by boosting neuronal regeneration, and synaptic repair. evidences have determined that IL-33 within the CNS can regulate inflammatory responses to facilitate neuroprotection, and regenerative processes, especially following neural damage, by boosting M2 macrophages, and regulatory Tregs to establish an anti-inflammatory environment.^[18]^

##### 3.5.3.2. Insights from animal model evidences

Evidences using animal models of depression have hence validated the mechanisms by which IL-33 operates. Literature hashighlighted that IL-33 performs a critical role in the progression of neuroinflammation, and depression. Particularly, IL-33 may impact the pathological processes of depression by regulating immune responses, and the release of inflammatory mediators. For example, IL-33 facilitates the generation of IL-10, an anti-inflammatory cytokine, signifying that IL-33 might mitigate depressive symptoms by restraining inflammatory responses.^[71]^

Besides, IL-33 interacts with other cytokines, such as IL-6, and IL-1β, which also play essential roles in the pathogenesis of depression. Evidences have uncovered that IL-33 expression is critically increased in mouse models of depression, and positively correlates with the severity of depressive behaviors.^[72]^ This phenomenon implies that IL-33 may increase depressive symptoms by boosting neuroinflammation.

In various depression animal models, the mechanisms of IL-33 action have been corroborated. In the chronic unpredicTABLE mild stress model, upregulation of IL-33 is linked to depressive behaviors in mice, and its suppressory impacts may be mediated via the regulation of the Janus kinase 2/signal transducer and activator of transcription 3 (STAT3) signaling pathway.^[72]^ This signaling pathway is considered a critical regulatory point in the neurobiological mechanisms of depression. Besides, IL-33 may contribute to the pathogenesis of depression by impacting neuronal survival, and apoptosis. Literature has confirmed that enhanced IL-33 levels are linked to neuronal apoptosis, likely worsening depressive symptoms.^[73]^ Hence, IL-33 not only performs a role in immune responses but may also take part in the onset of depression by influencing neuronal health.

Overall, evidences utilizing animal models of depression have revealed the critical role of IL-33 in neuroinflammation, and the pathophysiology of depression. Future research should hence investigate the potential of IL-33 as a therapeutic target, seeking to offer novel insights, and strategies for the treatment of depression.

### 3.6. Employment of IL-33 in clinical research

#### 3.6.1. Expression changes of IL-33 in depression patients

##### 3.6.1.1. Measurement, and results of IL-33 levels in clinical evidences

Numerous clinical literature has confirmed a critical decrease in IL-33 levels in both the blood, and cerebrospinal fluid of depression patients. This finding is linked to the pathological mechanisms of depression, reflecting that inflammation may play an essential role in the progression of the disorder. Literaturees stress that IL-33, as a cytokine, may play a central role in managing immune responses, and neuroinflammation. The decreased levels of IL-33 in depression patients may signify changes in their neuroinflammatory state, consistent with changes in other cytokines, such as IL-6, and IL-1β, which have also been explored in the pathogenesis of depression.^[74,75]^

In addition, evidences have demonstrated that IL-6, and interleukin-8 levels are critically increased in the cerebrospinal fluid of depression patients, which is tightly linked to the clinical manifestations of the disorder. The elevation of IL-6 may be linked to the exacerbation of depressive symptoms, signifying that CNS inflammatory responses may be vital in the pathogenesis of depression.^[76,77]^ These findings hence highlight the role of inflammation in depression, signifying that regulating the levels of these cytokines may supply new avenues for the treatment of depression.

Overall, the decreased levels of IL-33, and alterations in other inflammatory markers in depression patients confirm a complicated relationship between depression, and neuroinflammation. These discoveries offer important clues for identifying the biological basis of depression, and may offer new courses for future therapeutic strategies.

##### 3.6.1.2. Correlation analysis of IL-33 with depression severity

hence clinical evidences have assessed the relationship between diminished IL-33 levels, and the severity of depression, implying a limited correlation. This finding is consistent with numerous research outcomes, highlighting that IL-33 may be vital in the pathophysiological mechanisms of depression. For example, evidence has reported that IL-33 levels are critically increased in depression patients, and are linked to cognitive performance, signifying that IL-33 may have a regulatory role in the onset, and progression of depression.^[78]^ Besides, IL-33 is believed to take part in the inflammatory response of depression alongside other inflammatory markers, such as TNF-α, and IL-6, hence supporting the potential role of IL-33 as a biomarker in depression.^[79,80]^

In a study focusing on patients with chronic depression, researchers observed that IL-33 levels were negatively linked to the severity of depressive symptoms, signifying that IL-33 may play a protective role in the pathological mechanism of depression.^[81]^ In addition, changes in IL-33 levels may also be linked to alterations in other biomarkers, supplying new perspectives for given that’s tanding the complicated mechanisms of depression. via hence research, IL-33, and its related pathways may become novel targets for future depression treatments, especially in interventions targeting inflammatory responses. Overall, as a critical cytokine, the role of IL-33 in depression warrants in-depth investigation. Future studies should center on the interactions between IL-33, and other inflammatory factors, coupled with their dynamic changes at various stages of depression, aiming to offer new bases for the early diagnosis, and personalized treatment of depression.

#### 3.6.2. Potential of IL-33 as a biomarker

##### 3.6.2.1. Outlook of IL-33 in the diagnosis of depression

IL-33 has gained increasing attention as a potential biomarker for depression. evidences have confirmed that IL-33 levels are critically decreased in depression patients, and are negatively linked to the severity of the disorder, signifying that IL-33 may aid in analyzing the severity of depression.^[6]^ Besides, IL-33 performs a critical role in the immune response in psychiatric disorders, with evidences determining its alterations in depression coupled with other mental disorders, such as bipolar disorder, and schizophrenia.^[82]^ Besides, research stresses that differences in IL-33 levels, along with other neurotrophic factors, such as Mesencephalic Astrocyte-derived neurotrophic factor, can act as positive biomarkers to distinguish between depression patients, and healthy individoubles.^[83]^ These findings confirm that measuring IL-33 levels in blood or cerebrospinal fluid can supply more accurate diagnostics in clinical settings, especially in the early diagnosis, and subtyping of depression, which is of critical significance. Thus, IL-33 holds promise as a markedly sensitive, and specific biomarker for depression, aiding in increasing diagnostic accuracy, and efficiency.

##### 3.6.2.2. Potential of IL-33 levels as indicators of therapeutic response

Changes in IL-33 levels also hold potential as indicators of therapeutic response to antidepressant treatments. In clinical trials, patients undergoing antidepressant therapy typically exhibit a critical increase in IL-33 levels, which correlates with symptom alleviation.^[6]^ Hence, IL-33 can be applied not only for diagnosis, but also for observing treatment response, supporting clinicians in evaluating therapeutic efficacy, and modifying treatment regimens.^[84]^ This employment will offer important reference points for personalized treatment, increasing the precision, and positiveness of therapy.

#### 3.6.3. Progress in clinical trials linked to IL-33

##### 3.6.3.1. Recent therapeutic research, and progress targeting IL-33

Currently, therapeutic strategies targeting IL-33 chiefly center on the regulation of its receptor ST2, and the inhibition of IL-33 signaling pathways. Evidence has confirmed that IL-33 triggers following NFκB, and MAPK signaling pathways via the ST2 receptor, a mechanism that performs a critical role in various inflammatory diseases.^[51]^ Hence, anti-IL-33 monoclonal antibodies, and anti-ST2 monoclonal antibodies have become the main therapeutic strategies, with clinical evidences eurrently assessing the efficacy, and safety of these drugs in various diseases.^[85]^ Besides, IL-33 performs an critical role in the CNS, especially in managing neural plasticity, and inflammatory responses. Some evidences have uncovered that inhibition of IL-33 may help restore neural function, especially in the treatment of mood disorders, such as depression.^[6,43]^ These findings highlight the progression of IL-33 signaling pathway suppressors as a novel therapeutic method aimed at restoring neural plasticity, and emotional stability by regulating the pro-inflammatory, and anti-inflammatory functions of IL-33 (Table 1).

**Table 1:** Progress in clinical trials targeting IL-33, and its role in inflammatory diseases, and depression.

Research area	Target diseases	Research content	Clinical trial progress	References
Anti-IL-33, and anti-ST2 monoclonal antibodies	Different inflammatory diseases	Focuses on managing the IL-33 receptor ST2, and suppressing the IL-33 signaling pathway. IL-33 triggers following NFκB, and MAPK pathways via ST2, playing a critical role in various inflammatory diseases	Clinical trials are currently evaluating the efficacy, and safety of anti-IL-33 monoclonal antibodies, and anti-ST2 monoclonal antibodies in various diseases	^[51,85]^
Role of IL-33 in the CNS	Depression, mood disorders	IL-33 performs a critical role in the CNS, especially in managing neural plasticity, and inflammatory responses. Several evidences suggest that suppressing IL-33 may help restore neural function, especially in mood disorders like depression	Clinical evidences suggest that IL-33 may be involved in the pathogenesis of depression, especially in chronic stress or emotional stress models, by influencing microglial cells, and synaptic transforming	^[6,43]^
Employment of anti-IL-33 receptor antibodies in depression	Depression	IL-33, as an immunoregulatory factor, may play a role in depression by impacting microglial cells, and synaptic transforming. IL-33 is tightly linked to the onset, and progression of depression, especially in stress-related depression models	While clinical trials directly targeting anti-IL-33 receptor antibodies in depression are restricted, basic research, and clinical observations suggest IL-33’s involvement in depression	^[6,86]^
IL-33, and the blood–brain barrier	Depression	IL-33, as a large molecule, has difficulty crossing the blood–brain barrier, but its increase in cerebrospinal fluid is tightly linked to depressive manifestations, such as bipolar disorder, and postpartum depression	Increased IL-33 in cerebrospinal fluid is linked to various depressive manifestations, signifying that anti-IL-33 receptor antibodies could have antidepressant impacts by regulating the immune system, and increasing neurobiological changes	^[6]^

Anti-IL-33 =anti-interleukin-33 antibody, CNS =central nervous system, MAPK = mitogen-activated protein kinase, ST2 = suppression of tumorigenicity 2.

##### 3.6.3.2. Preliminary clinical trial results, and their clinical significance

The employment of anti-IL-33 receptor antibodies in depression has gained considerable attention. Although recent clinical trial results directly targeting anti-IL-33 receptor antibodies in depression patients are restricted, basic research, and clinical observations observe a potential association between IL-33, and the onset, and progression of depression. As a critical immunoregulatory factor, IL-33 may be involved in the pathological processes of depression by impacting microglial cells, and synaptic transforming in the brain.^[6,86]^ It is clearly apparent that a systematic review, and meta-analysis, have suggested a relationship between IL-33, and depression, especially in depression models linked to chronic stress or emotional stress, where IL-33 regulates neural progression, and synaptic function via interactions with microglial cells, thus reducing factors that induce depression.^[6]^ Although IL-33, as a large molecule, has difficulty crossing the blood–brain barrier, its increase in cerebrospinal fluid is tightly linked to various depressive manifestations, such as bipolar disorder, and postpartum depression.^[6]^ Thus, the employment of anti-IL-33 receptor antibodies in depression patients plays promise for regulating the immune system, increasing neurobiological alterations, and thus producing antidepressant impacts (Table 1).

### 3.7. Potential of IL-33 as a therapeutic target

#### 3.7.1. IL-33-targeted therapeutic strategies

##### 3.7.1.1. Recent status of anti-IL-33 receptor antibodies employments

Anti-IL-33 receptor antibodies, such as Tocilizumab, regulate the pro-inflammatory, and anti-inflammatory functions of IL-33 by blocking its interaction with the ST2 receptor. Clinically, Tocilizumab has been employed in the treatment of various inflammatory diseases, including rheumatoid arthritis, and systemic lupus erythematosus, among others.^[87]^ IL-33 displays pro-inflammatory impacts in various immune-mediated diseases, especially within tissues, such as the lungs, intestines, and brain, where it regulates immune responses while also involving regulatory functions.^[51,87]^ Tocilizumab displays critical anti-inflammatory impacts under the backdrop of suppressing IL-33 levels, and ameliorates inflammation-related symptoms. Preliminary evidences in the context of depression determine that Tocilizumab positively relieves depressive symptoms, and restores normal IL-33 expression levels.^[4,87]^ This finding offers a novel method for antidepressant therapy, especially targeting depression subtypes linked to neuroinflammation.

##### 3.7.1.2. Progression, and outlook of IL-33 signaling pathway suppressors

Beyond anti-receptor antibodies, suppressors targeting the IL-33 signaling pathway are actively given that progression. Literature highlights that the IL-33/ST2 signaling axis performs an essential role within the nervous system, and its inhibition may offer new therapeutic strategies for neurological disorders.^[10]^ These suppressors function by disturbing the interaction between IL-33, and the ST2 receptor or under the backdrop of suppressing following signaling pathways, thus reducing inflammatory responses, and boosting neural plasticity.^[43,65]^ Currently, research on IL-33 signaling pathway suppressors chiefly focuses on their employment in animal models, with preliminary results reporting that these suppressors can positively mitigate depression-like behaviors, and increase the functionality of neural networks.^[43,65]^ However, the clinical employment of these suppressors remains to be hence investigated, and validated.

#### 3.7.2. Clinical outlook, and challenges of IL-33-targeted therapy

##### 3.7.2.1. Advantages, and potential risks of IL-33 rherapeutic strategies

As a pivotal molecule in neuroimmune regulation, IL-33 boasts multifaceted regulatory mechanisms, thus demonstrating unique therapeutic potential in the targeted treatment of depression. The main strengths comprise IL-33’s ability to regulate neuroinflammatory responses, decrease or increase the expression of specific cytokines, and facilitate the synthesis of neurotrophic factors, thus increasing neural plasticity, and increasing neural function, and emotional states in depression patients.^[6]^ This composite mechanism may enable IL-33 to be vital in treating depression subtypes boosted by neuroinflammation. Besides, IL-33-targeted therapy allows for precise intervention in light of individouble pathological mechanisms. For example, in depression patients controlled by neuroinflammation, targeted modulation of IL-33 can support ameliorate the inflammatory state of the nervous system, supplying customized treatment regimens. Besides, by producing specific impacts on various depression subtypes, personalized treatment can be achieved, thus boosting therapeutic efficacy, and lowering side impacts.

However, the therapeutic potential of IL-33 is not without challenges. given thatlying risks comprise immune system side impacts, as IL-33 serves as a double-function factor within the immune system, likely disturbing immune balance, and causing immunoinhibition or overabundant immune responses. This imbalance could facilitate the risk of infections or cntribute to autoimmune diseases. In addition, the mechanisms of IL-33 within the nervous system are not yet fully revealed, especially its multifaceted functions within the CNS, which may cause complicated side impacts. Although its neuroprotective impacts have been preliminarily determined, long-term use of IL-33-targeted therapy might have unsupposed impacts on neural networks. Hence, hence animal studies, and clinical trials are necessary to analyze its long-term efficacy, and safety.

##### 3.7.2.2. Central issues to address in clinical translation

To advance the clinical employment of IL-33-targeted therapy, several central issues must be handled. Integrative assessment of efficacy, and safety is paramount. Although IL-33 has confirmed promising impacts in animal models, translating it into a extensively applicable clinical treatment regimen demands validation via multi-center, large-scale, and long-term clinical trials. These trials must not only verify the efficacy of IL-33-targeted therapy across various depression subtypes, but also center on its safety to ensure that it does not induce severe immunological or neurological side impacts. Concurrently, refining therapeutic strategies displays another challenge. The optimal dosage, administration route, and timing for IL-33-targeted therapy remain unclear, and call for hence refinement via preclinical, and clinical research to identify the most suiTABLE treatment protocols for depression patients. For example, whether to offer a single high-dose injection or phased dosing, and whether to combine it with other immunomodulatory treatments, necessitates systematic investigation.

In addition, the progression of biomarkers is essential in precision medicine, especially for predicting, and observing the efficacy of IL-33-targeted therapy. There is an urgent need to develop biomarkers that can precisely predict, and monitor therapeutic outcomes, thus boosting real-time adjustments to treatment plans to maximize efficacy, and minimize the risk of adverse reactions. Given that IL-33’s role spans immunology, neuroscience, and psychiatry, boosting its clinical translation necessitates interdisciplinary collaboration. Only via integrated, multidisciplinary research can the biological features of IL-33 be thoroughly revealed, and the most positive therapeutic strategies be formulated.

## 4. Conclusion, and outlook

### 4.1. Summary of contributions, and potential impacts

Our review mainly delves into the integrative role of IL-33 in neuroimmune regulation, HPA axis regulation, and neuroplasticity, leading it to a potential therapeutic target for depression. By managing the neuroinflammatory response, and boosting the expression of neurotrophic factors, IL-33 is likely to become an positive method for treating depression triggered by neuroinflammation. The investigation of IL-33 targeted therapy is integrative, especially for depression subtypes controlled by neuroinflammation, where IL-33 may become a revolutionary treatment strategy. Future research will hence center on the clinical efficacy of IL-33 targeted therapy, and offer more data for strengthening treatment strategies. As a new target for treating depression, IL-33 displays great potential. However, determining its prevalent clinical employment will call for developing basic research, and hence clinical trials. With a deeper given that standing of the mechanism of action of IL-33, it is supposed that more precise, and personalized treatment plans will be progressed for depression patients.

### 4.2. Challenges

Despite the enormous potential of IL-33 in depression research, there are still some challenges. Because of the incomplete determination of the specific mechanism of IL-33 in the nervous system, especially its regulatory role in neuroinflammation, and neuroplasticity, knowledge gaps, and technical barriers still exist. Besides, the interaction between IL-33, and other inflammatory cytokines has not been thoroughly explored, and given that standing its role in complicated pathological environments remains an urgent issue. Besides, the limitations of clinical research have also brought critical challenges. recent clinical evidence commonly involves small samples, and lack of diversity, which limits the universality of IL-33 targeted therapy. Future research must expand the sample size to ensure the inclusion of various patient populations of various ages, genders, and genetic backgrounds, in order to improve the integrative applicability of research results.

### 4.3. Future research courses

Future evidence should center on several central areas. It is essential to outline the specific role of IL-33 in various types of depression via carefully designed experiments, especially whether IL-33 displays various therapeutic impacts in acute stress-driven depression, and chronic neuroinflammation related depression. Besides, the mechanism of interaction between IL-33, and other inflammatory cytokines should be a concentration of future research. IL-33 does not act alone in the nervous system; It interacts complicatedly with other cytokines, such as IL-1β, and IL-6. Investigating the synergistic impacts of these cytokines will help to control potential therapeutic strategies. Besides, the integration of interdisciplinary strategies, and the employment of emerging technologies will play an essential role in developting IL-33 research. The immunomodulatory, and neuromodulatory functions of IL-33 call for interdisciplinary collaboration, including neuroscience, immunology, psychiatry, and molecular biology, to induce innovation, and translation of IL-33 targeted therapy strategies. Meanwhile, emerging technologies, such as single-cell sequencing, and brain imaging have enormous potential in IL-33 research. Single cell technology can precisely expound the role of IL-33 in various types of nerve cells, while brain imaging technology can monitor the real-time impacts of IL-33 targeted therapy on brain structure, and function.

## Author contributions

**Conceptualization:** Guiwei Wang, Xiaoming Zhang, Gang Duan, Shuping Yuan.

**Methodology:** Guiwei Wang, Xiaoming Zhang, Gang Duan, Shuping Yuan.

**Literature search:** Guiwei Wang, Xiaoming Zhang, Gang Duan, Shuping Yuan.

**Data analysis:** Guiwei Wang, Xiaoming Zhang, Gang Duan, Shuping Yuan.

**Writing – original draft:** Guiwei Wang, Xiaoming Zhang, Gang Duan, Shuping Yuan.

**Writing – review & editing:** Guiwei Wang, Xiaoming Zhang, Gang Duan, Shuping Yuan.
